# Co-Transcriptional Folding and Regulation Mechanisms of Riboswitches

**DOI:** 10.3390/molecules22071169

**Published:** 2017-07-13

**Authors:** Sha Gong, Yanli Wang, Zhen Wang, Wenbing Zhang

**Affiliations:** 1 Hubei Key Laboratory of Economic Forest Germplasm Improvement and Resources Comprehensive Utilization, Hubei Collaborative Innovation Center for the Characteristic Resources Exploitation of Dabie Mountains, Huanggang Normal University, Huanggang 438000, Hubei, China; shagong@hgnu.edu.cn; 2 Department of Physics, Wuhan University, Wuhan 430072, Hubei, China; 13212746736@163.com (Y.W.); zhenwang@whu.edu.cn (Z.W.)

**Keywords:** riboswitch, gene regulation, co-transcriptional folding

## Abstract

Riboswitches are genetic control elements within non-coding regions of mRNA. These self-regulatory elements have been found to sense a range of small metabolites, ions, and other physical signals to exert regulatory control of transcription, translation, and splicing. To date, more than a dozen riboswitch classes have been characterized that vary widely in size and secondary structure. Extensive experiments and theoretical studies have made great strides in understanding the general structures, genetic mechanisms, and regulatory activities of individual riboswitches. As the ligand-dependent co-transcriptional folding and unfolding dynamics of riboswitches are the key determinant of gene expression, it is important to investigate the thermodynamics and kinetics of riboswitches both in the presence and absence of metabolites under the transcription. This review will provide a brief summary of the studies about the regulation mechanisms of the *pbuE*, S_MK_, *yitJ*, and *metF* riboswitches based on the ligand-dependent co-transcriptional folding of the riboswitches.

## 1. Introduction

Riboswitches, as self-regulatory elements, regulate downstream gene expression through conformational changes driven by sensing specific metabolites [1,2,3,4,5,6], ions, and other physicalsignals [2,7]. Metabolite-specific riboswitches, the most widespread class that has been previously validated, are involved in many important biological processes, such as vitamin biosynthesis [8,9], nucleotide and amino acid metabolism [10,11], sulfur metabolism, and metal transport [1,12,13]. Most riboswitches are characterized by two domains: a most-conserved aptamer domain responsible for ligand binding by forming the aptamer structure, and an expression platform that converts folding changes in the aptamer domain into changes in gene expression [14,15,16]. The structure arrangement of riboswitches is able to modulate the expression of downstream coding sequences at the levels of transcription [17,18,19], translation [3,20], and RNA splicing [21,22]. However, there are several riboswitches, such as the S_MK_ riboswitch, that only utilize a single domain for both ligand binding and gene regulation [3,23].

**Figure 1:**
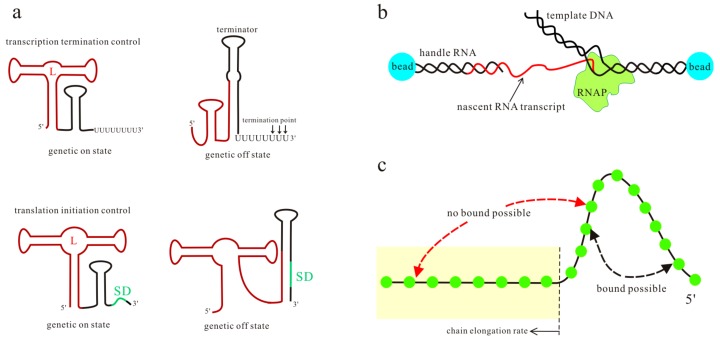
The transcription and translation control mechanisms of riboswitches (**a**), where the aptamer sequence and Shine–Dalgarno (SD) sequence are colored brown and green respectively; L is the ligand. A schematic of the optical-trapping assay and the kinetic Monte Carlo simulation is shown in (**b**,**c**). In (**c**), nucleotides (green circles) out of the tube (yellow) are possible to form base pairs. The kinetic Monte Carlo method grows the RNA chain with a constant rate, allowing more and more base pairs to form or open in the available sequence.

The commonly accepted molecular mechanism for riboswitch function proposes a signal-dependent RNA structural shift, usually between two distinct functional states, i.e., ligand bound state and unbound state (Figure 1a). One of the alternative states serves as the genetic off state, which may contain an intrinsic terminator hairpin (T hairpin) or a paired region covering the Shine-Dalgarno (SD) sequence [24,25,26,27]. The other state serves as the genetic on state that destroys these regulatory elements, and then the downstream genes are expressed. Translational control is critical for gene regulation in bacteria [28], while alternative pre-mRNA splicing is a central mode of gene regulation in eukaryotes [29]. For the riboswitches acting at the level of RNA splicing, the main difference in the two functional states is the structural flexibility near the relevant splice site [22,30]. Depending on ligand concentration and other intracellular factors, one of the two functional states is adopted by riboswitches to regulate downstream gene expression.

Previous studies have investigated many features of riboswitches to explore their regulation mechanisms, such as ligand recognition [8,31,32], folding landscape [3,33,34], the rates and energies for ligand binding and dissociation [19,24], and ligand specificity and its structural basis [35,36,37,38,39]. Experiments such as in-line probing [40,41,42,43], fluorescence spectroscopy [44,45,46], and single-round transcription assay [47,48], in conjunction with theoretical simulations [20,26,49], have established the basic principles that underpin riboswitch function in all three kingdoms of life. Most of these studies focus on the aptamer domain or the transcript products. However, in vivo nascent mRNAs fold as they are transcribed, and this sequential process affects the folding efficiency and the predominant structures. Thus, for some riboswitches, even though the high-resolution structures of the bound aptamer have been solved, the unbound functional structures formed during the transcription and the detailed regulation mechanisms are not well known [50,51,52,53]. As the ligand-dependent co-transcriptional folding and unfolding dynamics of riboswitches play an important role in exerting their functions [25,54,55], it is important to investigate the folding behaviors of riboswitches both in the presence and absence of metabolites in the transcription context. Recently, the optical-trapping assay (Figure 1b) has been developed to monitor co-transcriptional folding during the individual transcript of the *pbuE* adenine riboswitch [56]. Since the extension changes can be related to the number of nucleotides involved in the folding, by measuring the separation between beads, this assay furnishes a tool for probing structure formation and transition with force during the transcription. The ligand-dependent co-transcriptional folding of riboswitches has also been studied by using the kinetic Monte Carlo simulation to include RNA growth, by sequentially enlarging the subset of contacts that can be formed (Figure 1c) [54]. The effect of a bound ligand is mimicked by modifying the free energy stabilizing the multiloop region, but the method only considers the base pairs in the native structures. The analytical theory, which combines the master equation with the free energy landscape, has been proven to be a reliable tool to calculate co-transcriptional folding kinetics [57]. To explore the detailed regulation mechanisms of riboswitches, ligand binding kinetics have been incorporated into the theory [17]. By using this theory, the regulatory behaviors of several riboswitches under different transcription conditions have been investigated [17,58,59]. All of these studies produce a framework for describing the function of riboswitches at the system level. Here, we will provide a brief overview of the studies about the regulation mechanisms of the *pbuE*, S_MK_, *yitJ*, and *metF* riboswitches, based on their ligand-dependent co-transcriptional folding behaviors [17,58,59].

## 2. The Systematic Co-Transcriptional Folding Theory

From the early 1980s, key experiments showed that structure formation happens co-transcriptionally [60,61]. Many experiments have since substantiated this view [62,63,64]. For example, the co-transcriptional folding of the genomic ribozyme is assayed by monitoring the co-transcriptional self-cleavage of transcripts with variable lengths [65]. Likewise, a number of computational methods, such as RNAkinetics [66], Kinefold [67], and COFOLD [61], are developed to explicitly simulate co-transcriptional folding pathways of mRNAs as a series of structural changes over time. However, in order to investigate the riboswitch-mediated regulation mechanisms, ligand binding should be taken into account.

RNA’s secondary structure is stabilized mainly by the base-stacking interactions. As the rate for the formation of a base stack is usually larger than that of disrupting the stack, except the loop-closing stack under the folding condition, once the first few stacks in a helix are closed and stabilized, the zipping of the subsequent stacks in the helix would be fast [57]. It is proper to use the helices as building blocks for the study of the overall (slower) folding kinetics. The systematic helix-based computational theory, in which all structures are constructed according to helices as building blocks and a kinetic move is an addition or a deletion of a helix or an exchange between two helices, is successfully used to study the folding kinetics of the Hepatitis delta virus ribozyme and other mRNAs [57,68,69]. To simulate the co-transcriptional folding of mRNAs, this theory has been further developed to include RNA growth [70]. In this model, releasing one nucleotide by the transcription elongation complex to freely form structures is treated as one transcriptional step. If the transcription speed of an RNA sequence is ν nucleotides per second, the folding time window for each step will be 1/v seconds. From a time *t* when an *N*-nt chain is free to form structures to a time t+1/v when the (*N* + 1)-th nucleotide can freely form structures, the *N*-nt chain samples the conformation space at step *N*. Within this step, the population distribution is relaxed from ($p_{1}{\left(N\right)}_{\mathrm{begin}},\;p_{2}{\left(N\right)}_{\mathrm{begin}},\;....,p_{\Omega }{\left(N\right)}_{\mathrm{begin}}$] to [$p_{1}{\left(N\right)}_{\mathrm{end}},\;p_{2}{\left(N\right)}_{\mathrm{end}},\;....,\;p_{\Omega }{\left(N\right)}_{\mathrm{end}}$), where $p_{i}{\left(N\right)}_{\mathrm{begin}}$ and $p_{i}{\left(N\right)}_{\mathrm{end}}$ are the population of state *i* at the beginning and end of step *N*, respectively, and Ω is the number of conformations for the *N*-nt chain. This is defined as the *N*-th step. At each such transcriptional step, the population kinetics are calculated in the following manner: first, the conformation space is generated according to helices as building blocks, so a kinetic move is the addition or the deletion of a helix, or an exchange between two helices. Then, the transition rate $k_{i\to j}$ from state *i* to state *j* in the conformation space is calculated according to the structure relationship between the two states [57], whose free energies are obtained based on the nearest-neighbor model [71,72]. Finally, the population kinetics within the folding time window (1/v) are calculated by solving the master equation dp/dt=M⋅p, where the initial condition is determined by the final population distribution at the previous step [70]. M is the rate matrix with elements $M_{ij}=k_{i\to j}(i\neq j)$ and $M_{ii}=-\sum_{j\neq i}k_{i\to j}$, and p is the vector for the population distribution. For consecutive steps, the folding results of the current step turn into the initial condition of the next step. By applying this method from the first step to the end of the transcription, the co-transcriptional folding population kinetics of mRNAs can be calculated. To simulate transcription pausing at a specific site, a large number of effective time steps are assigned to the corresponding (paused) step. Co-transcriptional folding under different transcription rates can also be mimicked by changing 1/v.

Ligand binding kinetics are incorporated into the model to predict the ligand-dependent co-transcriptional folding behaviors of riboswitches [17]. As the ligand concentration is much larger than that of the mRNA in cells, the second-order ligand binding kinetics: mRNA+ligand → the ligand bound state, can be approximated as a linear relation. When the ligand is present, the ligand bound states are added to the conformation space. The ligand free state containing the aptamer structure can transit to the corresponding ligand bound state with the effective binding rate $k_{\mathrm{eff}}=k_{\mathrm{on}}[L]$, and the reverse transition with the rate $k_{\mathrm{off}}$, where [L] is the ligand concentration, and $k_{\mathrm{on}}$ and $k_{\mathrm{off}}$ are the experimentally measured association rate and dissociation rate, respectively. The ligand binding can stabilize the corresponding bound state by a free energy of $\Delta G_{\mathrm{binding}}=k_{B}T\ln(k_{\mathrm{on}}[L]/k_{\mathrm{off}})$, where $k_{B}$ is the Boltzmann constant, and T is the temperature. We have studied the co-transcriptional folding behaviors of the *pbuE* [17], S_MK_ [58], *yitJ*, and *metF* riboswitches by using this method [59].

## 3. The Kinetic Regulation Regime of the *pbuE* and *metF* Riboswitches

**Figure 2:**
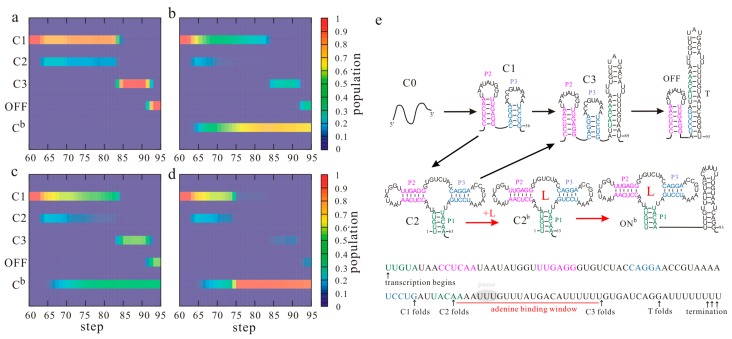
The ligand-dependent co-transcriptional folding behaviors of the *pbuE* riboswitch. The population kinetics of main structures from step 60 under different transcription conditions are shown: 20 nt/s, 0 µM adenine (**a**); 20 nt/s, 100 µM adenine (**b**); 50 nt/s, 100 µM adenine (**c**); 50 nt/s, 100 µM adenine with pausing (60 s) at step 67 (**d**). The superscript “b” denotes the corresponding state with the ligand bound and C^b^ denotes the ensemble of all the bound states. Main co-transcriptional folding pathways and folding events are mapped in (**e**). T is the terminator hairpin; L is the ligand (adenine). Nucleotides within paired regions of the aptamer structure are colored differently.

Among the riboswitches known to date, the *pbuE* adenine riboswitch is a rare type of “on” switch that activates gene expression upon ligand binding by controlling transcription termination [55]. Its aptamer structure is characterized by a three-way junction consisting of helices P1, P2, and P3, which are further stabilized by tertiary interactions in the folded state. The full-length *pbuE* riboswitch has been found to be fixed at a ligand-incompetent structure [24,55], and the aptamer structure is not formed during the refolding process [17], suggesting that the transcription context plays a key role in its function. The folding events of the *Bacillus subtilis pbuE* riboswitch during the transcription were monitored by the helix-based co-transcriptional folding method [17]. Figure 2 shows the population kinetics of the main structures (a–d) and folding pathways (e) under different transcription conditions. The folding of helices P2 and P3, which occurs in turns, is not affected by the transcription conditions. However, when helix P1 is nucleated, adenine can bind to the riboswitch. Thus, the population kinetics of the main states and folding pathways from step 60 are shown in Figure 2 for further discussion. As state C1, consisting of helices P2 and P3, is the most stable state before the expression platform is transcribed, almost all the riboswitches populate at this state at step 60 (Figure 2a). When helix P1 is nucleated from step 62, state C1 can transit to the aptamer structure C2 by forming helix P1 with the rate $k_{C1\to C2}=1.1\times 10^{3}s^{-1}$. As forming this short nonlocal helix needs to close the big multiloop, which leads to an increase in the free energy ($\Delta G_{C1}=-12.42\;\mathrm{kcal}/\mathrm{mol}$, $\Delta G_{C2}=-11.69\;\mathrm{kcal}/\mathrm{mol}$), only about one fifth of the RNAs equilibrate into state C2. In the absence of adenine, the aptamer structure C2 will be gradually invaded by the T hairpin to form state C3 with elongation of the nascent RNA chain. At the end of the transcription, about 98% of the riboswitches fold through C3 to the OFF state with the T hairpin, which prompts transcription release at the terminator U tract (termination point) [14,73]. Given that factors such as RNA polymerase (RNAP) and nucleoside triphosphate (NTP) may affect the termination efficiency (TE) [74,75], the result agrees well with a TE of 89% given by the gel-based assay [56].

Conversely, when the ligand is present, adenine binding stabilizes the aptamer structure C2 to form the bound state C2^b^ (the superscript “b” denotes the state with ligand bound, Figure 2e) by introducing tertiary interactions. With the growth of the nascent RNA chain, the bound aptamer will prevent the formation of the T hairpin. Instead, it will direct the folding of the ligand bound ON state (ON^b^) by forming a hairpin, which can introduce about −1.61 kcal/mol free energies. The results in Figure 2b suggest that with 100 µM adenine, about 67% of the riboswitches populate at the ON^b^ state at the end of the transcription. Even if adenine binding would contribute a free energy as low as (4 ± 1) kcal/mol [76], this state is still much more unstable than the OFF state ($\Delta G_{\mathrm{OFF}}=-29.33\;\mathrm{kcal}/\mathrm{mol}$, $\Delta G_{\mathrm{ON}}=-13.30\;\mathrm{kcal}/\mathrm{mol}$). However, due to the slow dissociation rate of the bound aptamer, the RNAP is able to successfully pass the termination signal before the ON^b^ state equilibrates into a low-energy OFF state, consistent with the riboswitches operating under a kinetic regulation regime. Hence, the formation of the two functional states (ON^b^ and OFF) are both irreversible events for effective riboswitch-mediated gene regulation.

During the transcription process, the aptamer structure is formed before the OFF state. In the absence of the ligand, the aptamer structure would quickly transit to the OFF state. However, in the presence of the ligand, adenine can bind to the aptamer structure and then preclude the structure from forming an OFF state. The ligand binding must occur during the transcription process, and the action of the riboswitch is sensitive to co-transcriptional folding. During the transcription, the time window allowed for adenine binding is from step 62 when the aptamer structure can be formed to step 84 when state C3 becomes much more stable ($\Delta G_{C3}=-22.98\;\mathrm{kcal}/\mathrm{mol}$). This binding time window provides the riboswitch with only one chance to switch genetic on or off. Excepting the termination region, there is a short series of uridine (U) residues within the template, which may induce transcription pausing at around step 67 [24,55], considering about 12 nt enclosed by the RNAP [77,78]. If the pause duration is τ s, the time $t_{\mathrm{bind}}$ allowed for adenine binding will be (22/v+τ) s, where v is the transcription rate. When the transcription rate increases from 20 nt/s to 50 nt/s, the population of the ON^b^ state decreases from 67% (Figure 2b) to 43% (Figure 2c) with 100 µM adenine. Nevertheless, even under the same conditions, the population of the ON^b^ state will increase to about 86% if the transcription pause occurs (Figure 2d). Obviously, a fast transcription rate yields a short binding time $t_{\mathrm{bind}}$, thereby repressing the formation of the bound state, whereas the transcription pausing has the opposite effect. In other words, the ligand concentration, the transcription speed, and pausing are coupled to modulate the *pbuE* riboswitch to perform regulatory activities. Since the transcription rate and pausing are affected by intracellular factors such as NTP concentration and proteins [19,79,80], the expression of the *pbuE* riboswitch downstream gene is largely dependent on the intracellular environment.

**Figure 3:**
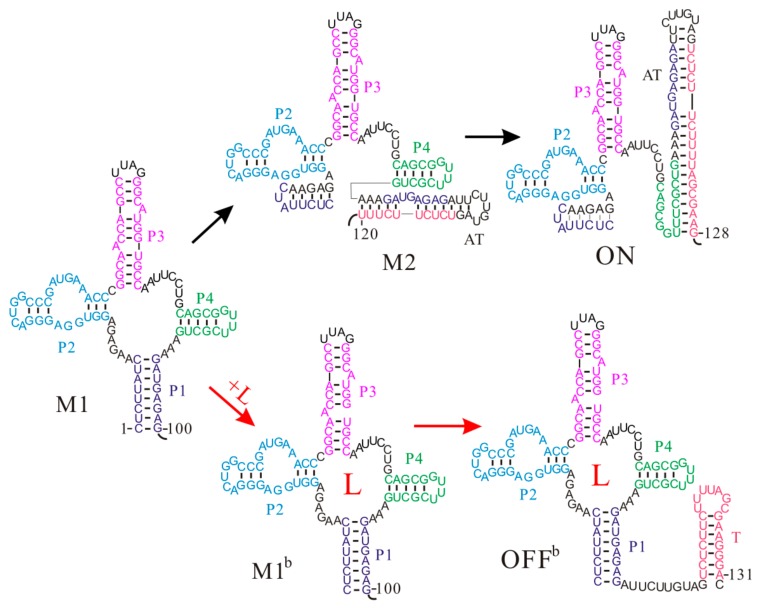
The main co-transcriptional folding pathways of the *metF* S-box riboswitch. Helices P1, P2, P3, and P4 and the T hairpin are colored differently. AT is the anti-terminator hairpin; L is the ligand S-adenosylmethionine (SAM).

Even though the aptamer structure of the S-box riboswitches is a four-way junction structure, the *Thermoanaerobacter tengcongensis metF* S-box riboswitch shows similar co-transcriptional folding behaviors to that of the *pbuE* riboswitch [59]. Once the aptamer sequence is released during the transcription, the aptamer structure M1 consisting of helices P1, P2, P3, and P4 is formed without any trapped states (Figure 3). As the nascent RNA chain grows, the upper part of the anti-terminator (AT) hairpin nucleates and gradually invades into the aptamer structure M1. Soon, this structure will be replaced by state M2, in which helix P1 is disrupted. By the time the RNAP reaches the termination point, a low-energy ON state with the AT hairpin is fully formed, and at the same time helix P4 is broken as well ($\Delta G_{\mathrm{ON}}=-48.88\;\mathrm{kcal}/\mathrm{mol}$, $\Delta G_{\mathrm{OFF}}=-42.58\;\mathrm{kcal}/\mathrm{mol}$). In this case, the RNAP can run through to the end of the template and the gene is expressed. On the contrary, if the ligand S-adenosylmethionine (SAM) successfully binds to the aptamer structure before the expression platform is transcribed, the bound aptamer M1^b^ will fold to an OFF^b^ state by directly forming the T hairpin. Due to the long half-life of the SAM-RNA complex [47], the transcript has been terminated before the riboswitch folds into the thermodynamically favored ON state [59], suggesting a kinetic regulation regime. Since the formation of the ON state disrupts the aptamer structure and it is very stable, SAM binding only can occur before formation of this state. This demonstrates that the ligand binding window is limited during the transcription process, and the fate of downstream gene expression depends on the folding of the riboswitch within this time period. Thus, the regulatory activities of the *metF* riboswitch are also tied to the intracellular environment. In addition, although both states M2 and ON have been proposed as the genetic on state of the *metF* riboswitch previously [52,53], our results suggest that state M2 is just an intermediate structure and persists for several transcriptional steps, in agreement with the recent study [51].

## 4. The Association of Thermodynamic and Kinetic Regulation Regimes of the *yitJ* Riboswitch

**Figure 4:**
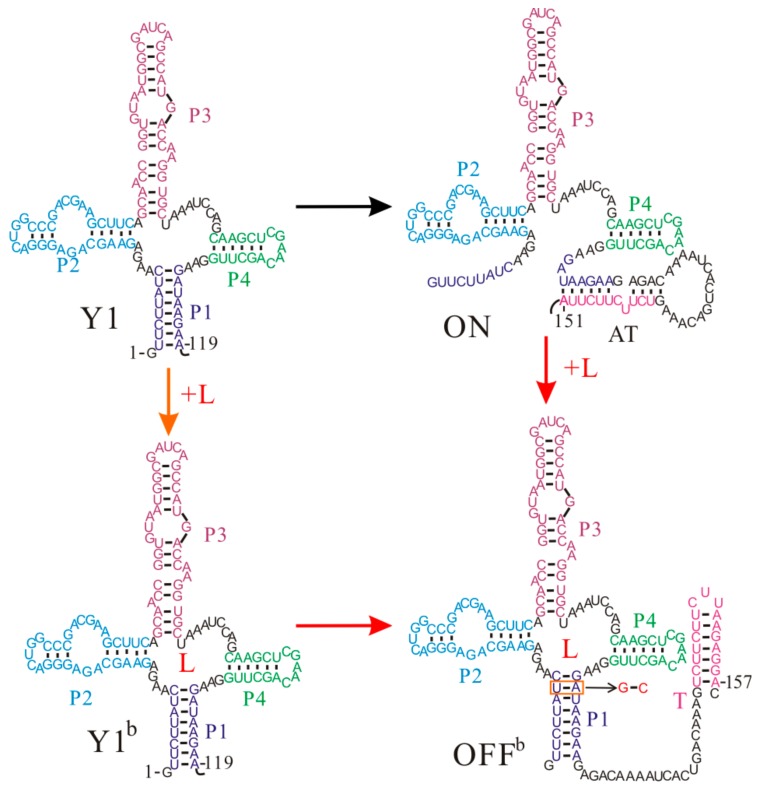
The main co-transcriptional folding pathways of the *yitJ* S-box riboswitch. helices P1, P2, P3, P4 and the T hairpin are colored differently. L is the ligand SAM. The AU base pair depicted in the red box within OFF^b^ state denotes the mutated base pair (GC).

The *Bacillus subtilis yitJ* riboswitch is another SAM-specific riboswitch that has been intensively studied [13,81]. The two S-box (*yitJ* and *metF*) riboswitches have similar OFF state structures and share some features, such as the reliance of termination efficiency on SAM concentration, but their regulation mechanisms are different [59]. The aptamer structure Y1 is formed as soon as the relevant nucleotides are synthesized. When SAM is present at sufficient concentrations, SAM binding stabilizes the aptamer structure Y1, which induces the formation of the T hairpin (OFF^b^) as the nascent RNA grows (Figure 4) [59]. However, if the ligand cannot bind to the riboswitch before the expression platform is synthesized, the AT hairpin (ON) will be formed instead, which allows the RNAP to successfully pass the termination point. As the formation of the AT hairpin only disrupts helix P1 in the aptamer structure, an ON state can quickly equilibrate into an OFF state ($k_{\mathrm{ON}\to \mathrm{OFF}}=8.9\times 10^{2}s^{-1}$) through the tunneling pathway within one transcriptional step. This implies that their population distribution is thermodynamically controlled when the RNAP reaches the termination point. In addition, because the two states ($\Delta G_{\mathrm{ON}}=-41.27\;\mathrm{kcal}/\mathrm{mol}$, $\Delta G_{\mathrm{OFF}}=-40.10\;\mathrm{kcal}/\mathrm{mol}$) have similar free energies, a single base pair mutation by changing UA to GC in helix P1, can result in high termination efficiency even without the ligand [13], suggesting thermodynamic control characteristics.

Since the transition from an OFF state containing the aptamer structure to an ON state is very fast, even if the *yitJ* riboswitch predominantly folds into an ON state during the transcription, this fold is in equilibrium with a small population of the OFF state. Upon SAM binding, the equilibrium is shifted toward the thermodynamically favored OFF^b^ state. The free energy of the ON state is about −1.17 kcal/mol lower than that of the OFF state, and only about 0.13 µM ligand is required to make an OFF^b^ state slightly more stable than an ON state ($\Delta G_{\mathrm{binding}}=-1.19\;\mathrm{kcal}/\mathrm{mol}$, $k_{\mathrm{on}}=8.6\times 10^{4}M^{-1}\;s^{-1}$, $k_{\mathrm{off}}=0.0016s^{-1}$) [47,59]. However, the ligand binding is a kinetic process, and whether it reaches equilibrium depends on the binding time window and the effective binding rate ($k_{\mathrm{eff}}=k_{\mathrm{on}}[L]$). For this riboswitch, the ligand binding could occur from the 117th step when the aptamer structure is formed to the termination point. At the end of the transcription, the ligand bound states can occupy about $p_{\mathrm{bound}}=p_{\mathrm{eq}}(1-\exp(-k_{\mathrm{on}}[L]\times 40/v))$ of the population, where $p_{\mathrm{eq}}$ is the equilibrium distribution of the ligand bound states. Even at 3 μM SAM, an OFF^b^ state only can obtain about 11% of the population at 50 nt/s, far less than the equilibrium value $p_{\mathrm{eq}}=94.8\%$ [59], because of the slow effective binding rate. Namely, a much higher ligand concentration may be required to trigger riboswitch regulation relative to the dissociation constant. Additionally, the amount of the ligand required to trigger the switch is dependent on the transcription rate. Thus, the transcription process should also be determinant for riboswitch activation. All these results strongly suggest the *yitJ* riboswitch operates under a combination of thermodynamic and kinetic regulationmechanisms [59].

## 5. The Thermodynamic Regulation Regime of the S_MK_ Riboswitch

**Figure 5:**
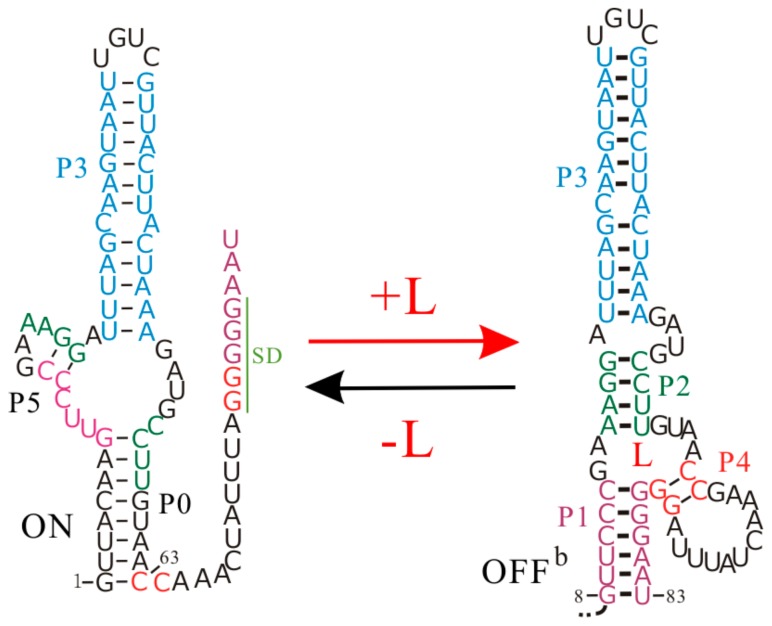
The regulation mechanisms of the S_MK_ riboswitch. L is the ligand SAM.

The *Enterococcus faecalis* SAM-III (S_MK_) riboswitch is one of five distinct classes of SAM-specific riboswitches that have been discovered [35]. This riboswitch senses the intracellular SAM or its derivative *S*-adenosylhomocysteine to regulate the translation of the metK gene [36,82], which encodes the synthetase of SAM, a cofactor in the methylation reactions of proteins, nucleic acids, and other biomolecules [82,83]. It is a typical riboswitch that operates at the translation level, and to be more specific, its ligand binding and regulatory domains are coincident [3,26]. The SD sequence directly takes part in binding SAM, and is sequestered by base pairing with the anti-SD sequence in the presence of the ligand (Figure 5).

The co-transcriptional folding and refolding behaviors of the S_MK_ riboswitch as well as its shortened construct have been studied by the recently developed method [58]. In contrast to most riboswitches containing two separate domains, this riboswitch, with a single domain to perform gene regulation, displays many unique features in its regulation activities. During the free folding and co-transcriptional folding processes without SAM, the S_MK_ riboswitch quickly folds into the low-energy ON state ($\Delta G_{\mathrm{ON}}=-18.80\;\mathrm{kcal}/\mathrm{mol}$), while the SAM binding pocket structure (OFF state) consisting of helices P1, P2, P3, and P4 is not formed. As a co-transcriptional fold follows a sequential process, an ON state with the unstructured SD sequence is fully formed (step 63) prior to an OFF state (step 83) during the transcription (Figure 5), and it is more stable than the OFF state ($\Delta G_{\mathrm{OFF}}=-17.40\;\mathrm{kcal}/\mathrm{mol}$). Thus, even with SAM at a saturating level, the S_MK_ riboswitch still first folds to an ON state, instead of the binding pocket structure OFF, which is formed near the end of the transcription. For this riboswitch, whether the ligand is present or not, the main co-transcriptional folding pathway is the same. Unlike the *pbuE* and two S-box riboswitches, sequential folding during the transcription process is not necessary for the SAM-III riboswitch to efficiently perform gene regulation.

Although an ON state is first formed, it will fold into the bound state OFF^b^ when SAM is abundant, since the ligand binding can make the OFF^b^ state more stable than the ON state. The transition from ON state to OFF state requires it to undergo conformational rearrangements, as it needs to break helices P0 and P5 and form helices P1, P2, and P4. Compared to the mRNA degradation rate ($3\;\min^{-1}$) [26], the tunneling pathway from an ON state to an OFF state yields a faster transition rate ($4\times 10^{-2}s^{-1}$). If SAM binding to the full-length riboswitch is described as a three-state transition model: $\mathrm{ON}↔\mathrm{OFF}+\mathrm{SAM}↔{\mathrm{OFF}}^{b}$, the population of ON and OFF^b^ states will be thermodynamically distributed [58]. Its switch efficiency is linked to the stability of the two function structures instead of the transcription context. Therefore, the S_MK_ riboswitch can quickly switch back and forth to regulate the translation of its host gene in response to the ligand concentration, strongly supporting a pure thermodynamic regulation mechanism.

The thiamine pyrophosphate (TPP) riboswitch within the *NMT1* gene from *Neurospora crassa* is a typical riboswitch that controls RNA splicing [84]. The TPP riboswitch aptamer forms a tuning fork architecture, in which the prongs are formed by two parallel stacking helices and arranged via a central three-way junction [85,86,87]. TPP binding stabilizes the aptamer structure, increasing the structural flexibility near the 5′ splice site. The effect of the change yields the long spliced mRNAs carrying the short upstream open reading frames that compete with the translation of the main open reading frame. In the absence of TPP, the riboswitch adopts an alternative structure that occludes the 5′ splice site, leading to a short spliced mRNA, and then inducing the expression of the *NMT1* gene. This riboswitch also utilizes a single domain for both ligand binding and gene regulation, so we can speculate that the S_MK_ and TPP riboswitches may share some common regulatory features.

## 6. Conclusions

Riboswitches, which adopt alternative RNA folds to regulate gene expression, are sensitive to co-transcriptional folding events. Co-transcriptional folding following a sequential progression, with associated structures forming within seconds (or less) of the times that their sequences clear the RNAP footprint, poses a serious challenge in the quest for detecting these structures in an experimental context. By incorporating the ligand binding kinetics into the helix-based co-transcriptional folding theory, the regulation mechanisms of the riboswitches can be attained through simulating the co-transcriptional folding behaviors of the riboswitches in the presence/absence of ligands under different conditions. The good agreement of the results with the experiments demonstrates that it is a reliable tool to study regulation mechanisms for riboswitches and the functions of other RNAs.

For the riboswitches under the same regulation mechanisms, their regulatory behaviors and functional states exhibit many common features. The kinetically controlled riboswitches, such as the *pbuE* and *metF* riboswitches, usually exert regulatory control of transcription. Their ON states are much more stable (unstable) than their OFF states, so the ligand binding does not offset the free energy difference even at saturating concentrations. In addition, because the formation of the most stable functional state, such as the OFF state of the *pbuE* riboswitch, largely disrupts the aptamer structure, the full-length riboswitches often resist the ligand binding. Hence, the ligand binding is linked to the transcription context and it must occur prior to the formation of this stable state during the transcription. That is to say, the time window allowed for the ligand binding is limited. This limited time window can be modulated by the transcription process. For example, proper transcription pausing and slow elongation rates could give the aptamer structure more time to bind to the ligand. For these riboswitches, even the bound functional state, such as the ON^b^ state of the *pbuE* riboswitch, is not the most stable state, and it will remain folded before the RNAP has made the genetic decision because of the slow dissociation rate. Therefore, the riboswitches are always irreversible genetic switches, and their intracellular environments play a key role in their regulation; this has also been found in other systems [19].

Co-transcriptional folding is fundamental for kinetically controlled riboswitches to exert modulatory effects far from equilibrium. Conversely, for the riboswitches operating under a pure thermodynamic regime, such as the S_MK_ and *add* adenine riboswitches, the transcription context is not required for riboswitch function, as the ligand can bind post-transcriptionally [25,58,88]. These riboswitches are likely to function at the translation level. As their unbound functional states allow a large portion of the aptamer structures to form, the responsiveness of the full-length riboswitches to the ligand can be retained. Contrary to kinetically controlled riboswitches, the ON and OFF states of these riboswitches have similar free energies and much faster transition rates compared to the mRNA degradation rate. Thus, the two functional states, such as the ON and OFF^b^ states of the S_MK_ riboswitch, are thermodynamically distributed. The riboswitches reversibly interconvert between the two functional states, with the outcome ultimately determined by thermodynamic changes due to ligand binding. For the *yjtJ* riboswitch acting at the transcription level, its ON and OFF states have similar free energies and fast transition rates. However, the amount of the ligand required to trigger the switch is not only dependent on the dissociation constant, but also on the transcription process, so it functions under an association of thermodynamic and kinetic regimes.
